# A Fine-Tuned Bidirectional Encoder Representations From Transformers Model for Food Named-Entity Recognition: Algorithm Development and Validation

**DOI:** 10.2196/28229

**Published:** 2021-08-09

**Authors:** Riste Stojanov, Gorjan Popovski, Gjorgjina Cenikj, Barbara Koroušić Seljak, Tome Eftimov

**Affiliations:** 1 Faculty of Computer Science and Engineering Ss Cyril and Methodius, University- Skopje Skopje the Former Yugoslav Republic of Macedonia; 2 Computer Systems Department Jožef Stefan Institute Ljubljana Slovenia; 3 Jožef Stefan International Postgraduate School Ljubljana Slovenia

**Keywords:** food information extraction, named-entity recognition, fine-tuning BERT, semantic annotation, information extraction, BERT, bidirectional encoder representations from transformers, natural language processing, machine learning

## Abstract

**Background:**

Recently, food science has been garnering a lot of attention. There are many open research questions on food interactions, as one of the main environmental factors, with other health-related entities such as diseases, treatments, and drugs. In the last 2 decades, a large amount of work has been done in natural language processing and machine learning to enable biomedical information extraction. However, machine learning in food science domains remains inadequately resourced, which brings to attention the problem of developing methods for food information extraction. There are only few food semantic resources and few rule-based methods for food information extraction, which often depend on some external resources. However, an annotated corpus with food entities along with their normalization was published in 2019 by using several food semantic resources.

**Objective:**

In this study, we investigated how the recently published bidirectional encoder representations from transformers (BERT) model, which provides state-of-the-art results in information extraction, can be fine-tuned for food information extraction.

**Methods:**

We introduce FoodNER, which is a collection of corpus-based food named-entity recognition methods. It consists of 15 different models obtained by fine-tuning 3 pretrained BERT models on 5 groups of semantic resources: food versus nonfood entity, 2 subsets of Hansard food semantic tags, FoodOn semantic tags, and Systematized Nomenclature of Medicine Clinical Terms food semantic tags.

**Results:**

All BERT models provided very promising results with 93.30% to 94.31% macro F1 scores in the task of distinguishing food versus nonfood entity, which represents the new state-of-the-art technology in food information extraction. Considering the tasks where semantic tags are predicted, all BERT models obtained very promising results once again, with their macro F1 scores ranging from 73.39% to 78.96%.

**Conclusions:**

FoodNER can be used to extract and annotate food entities in 5 different tasks: food versus nonfood entities and distinguishing food entities on the level of food groups by using the closest Hansard semantic tags, the parent Hansard semantic tags, the FoodOn semantic tags, or the Systematized Nomenclature of Medicine Clinical Terms semantic tags.

## Introduction

Food is one of the most important environmental factors that affects human health [1]. However, even healthy and ecofriendly foods can cause health problems when consumed together with specific drugs or while having specific diseases. Comprehensive dietary assessments are required to understand how food influences our health, after considering various aspects. Automating the detection of food entities is important for several applications such as food-drug interactions and health issues related to food.

Computer science can greatly contribute to this research topic, especially in the areas of machine learning, natural language processing (NLP), and data analysis. Data collected in studies carry important information, which is not easily extracted when it has been gathered from different data sources. The main problem is that these data are presented in different formats: structured, semistructured, and unstructured. Additionally, the data consist of entities from different domains such as food and nutrition, medicine, pharmacy, ecology, and agriculture. The extraction of this information allows the creation of knowledge graphs [2], which represent a collection of interlinked descriptions of entities—objects, events, or concepts—by using semantic metadata and providing a framework for data integration, unification, analytics, and sharing.

**Figure 1:**
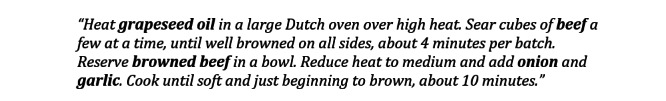
Recipe example.

To create a knowledge graph, first, we should have methods that can be used for information extraction, which is the task of automatically extracting structured information from unstructured textual data. In most cases, information extraction is performed by using named-entity recognition (NER) methods (ie, a subtask of information extraction), which deal with automatically detecting and identifying phrases (ie, one or more words [tokens]) from the text that represents the domain entities. Let us assume the following recipe example (Figure 1):

The phrases in bold (Figure 1) are the named entities that should be recognized in the process of information extraction, and they should be linked to their corresponding domain entity tag. In the simplest case, they may be linked to the generic “Food” class, but extracting the more specific food class by a level of food group may be of higher value, because this class may potentially provide multiple nutrition facts that may allow new use cases such as ingredient substitution.

Several types of NER methods exist depending on their underlying methodology: (1) *dictionary-based* [3], which return only entities that are mentioned in the dictionary in which they are based; (2) *rule-based* [4,5], which use a dictionary in combination with rules that describe the characteristics of the entities in the domain of interest; (3) *corpus-based* [6,7], which learn a supervised machine learning model by using an annotated corpus; (4) *active learning–based* [8], which use semisupervised learning to train a model that does not require a large annotated corpus but instead interacts with the user to query for new annotations that are used for iteratively improving the model; and (5) *deep learning–based* [9], which use deep neural networks to train a model that requires a large amount of annotated data. Nowadays, fine-tuning the bidirectional encoder representations from transformers (BERT) [10] provides state-of-the-art results in NER tasks. However, the task of fine-tuning the BERT model for NER requires a domain-specific annotated corpus.

In the past 2 decades, a large amount of work has been done to address this problem in the biomedical domain [11-17]. All of this work is supported by the existence of diverse biomedical vocabularies and standards such as the Unified Medical Language System [18], together with the collection of a large amount of annotated biomedical data (eg, in the domain of drugs, diseases, and other treatments) from numerous biomedical NLP workshops [19-26]. The existence of such resources and information extraction methods allows the creation of knowledge graphs that can support the biomedical domain and clinical practices [27,28].

In contrast to the biomedical domain, the food domain is relatively inadequately resourced. There are few semantic models (ie, ontologies) [29], each of which has been developed for very specific applications. One such example is the Ontology for Nutritional Epidemiology, which was developed to describe dietary food assessment [30]. Until recently, there was no annotated food corpus, which meant that the available food NERs were rule-based. Hanisch et al [4] presented a rule-based NER known as drNER for information extraction from evidence-based dietary recommendations. Food entities are among the domain entities of interest that are extracted. However, drNER extracts several food entities as one. This was improved by developing the rule-based NER Food Information Extraction [31], where the rules incorporate computational linguistics information in combination with food semantic annotations from the Hansard corpus [32]. Another way to perform food information extraction is to use the NCBO (National Center for Biomedical Ontology) annotator [33], which is a web service that annotates text by using food ontology concepts that are part of the BioPortal software services [34]. It can be combined with the following ontologies: FoodOn [35], OntoFood, and SNOMED CT (Systematized Nomenclature of Medicine Clinical Terms) [36]. A comparison of 4 NER methods (Food Information Extraction, NCBO [SNOMED CT], NCBO [OntoFood], and NCBO [FoodOn]) is presented by Popovski et al [37], who showed that Food Information Extraction provides the best results in distinguishing food from nonfood entities. The main weakness of the abovementioned NERs is that they all depend on other external resources such as taxonomies, ontologies, or previously developed annotators, which further can be a problem if some of the resources become inaccessible. This also opens new directions for future research regarding the development of more robust food NERs.

At the end of 2019, an annotated food corpus known as FoodBase [38] was published. The ground truth corpus consists of 1000 recipes, where for each recipe, the food entities mentioned in it are first extracted and then annotated using the hierarchical Hansard food semantic tags (eg, AG.01 [food], AG.01.h.02 [vegetables], AG.01.h.02.i [herb], AG.01.n.15 [pastry], AE.10 [fish]). The corpus is organized according to the BioC format, which is a minimalist approach for interoperability for biomedical text processing [39]. The availability of the FoodBase corpus allowed the development of the first food corpus-based NER known as bidirectional long short-term memory for food named-entity recognition (BuTTER) [40], where bidirectional long short-term memory (BiLSTM) in conjunction with conditional random fields (CRFs) and different representation learning methods have been explored to develop NER that distinguishes between food versus nonfood entities. In addition to this, the FoodOntoMap resource was published [41], where for the same entities found in FoodBase, the semantic tags from FoodOn, OntoFood, and SNOMED CT were assigned. With this, the food entities were normalized to different food semantic resources, which additionally links the food semantic resources.

**Figure 2:**
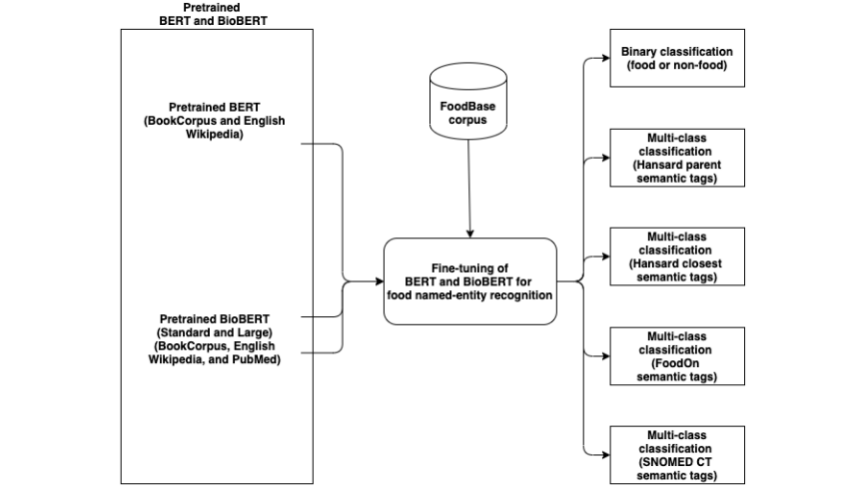
Food named-entity recognition flowchart. BERT: bidirectional encoder representations from transformers; NER: named-entity recognition; SNOMED CT: Systematized Nomenclature of Medicine Clinical Terms.

Enabled by the availability of several food resources that were published toward the end of 2019, we introduce a fine-tuned BERT model that can be used for food information extraction, called as FoodNER. BERT is known to achieve state-of-the-art results in NER tasks [42-44], and hence, we utilize it to develop a more robust model for food information extraction. The flowchart of FoodNER is presented in Figure 2. It is developed using a predefined BERT model, which can be the original BERT or some variation of BioBERT. Using them, fine-tuning is performed on the FoodBase corpus to address several different tasks: food or nonfood entity and 4 types of distinguishing food entities, depending on the semantic resource from where the semantic tags are taken (ie, Hansard semantic taxonomy [done twice on different hierarchical levels from the taxonomy], FoodOn, and SNOMED CT).

The main contributions of this study are as follows:

1. We fine-tuned different BERT models on different semantic resources from which the food semantic tags are taken. All BERT models have very promising results, obtaining around 73.39%-78.96% macro F1 score. All in all, it represents the new state-of-the-art in food information extraction.
2. In comparison with the already existing food rule–based (Food Information Extraction) and corpus-based (BuTTER) NER methods regarding the task of distinguish between food or nonfood entity, FoodNER provides similar results. However, it is more robust than the rule-based approaches since it does not require the continuous availability of additional external resources, which can be a problem regarding sustainability. Additionally, comparing it to the corpus-based method BuTTER, it is the first model that can predict food groups instead of just distinguishing between food versus nonfood entities.
3. The source code used for fine-tuning the different FoodNER models is publicly available. All models are also included in FoodViz [45], which is a new tool for the visualization of food annotations in text. The users can additionally select which model they want to use and annotate their data.

In this study, we used the FoodBase ground truth corpus for building and evaluating FoodNER models for distinguishing food versus nonfood entity as well as for distinguishing food entities concerning the Hansard semantic tags. The BuTTER approach is used as baseline for comparing the performance of the FoodNER models. The FoodOntoMap extension of the FoodBase ground truth corpus is also used for training and evaluating the FoodNER models concerning the SNOMED CT and FoodOn semantic tags.

## Methods

### FoodBase Data Corpus

The FoodBase data corpus is a recently published corpus with food annotations [38]. It consists of 2 versions: curated and uncurated. The curated version consists of 1000 recipes that are annotated using a rule-based NER and then manually checked by subject matter experts who removed the false positives and added the false negatives to create a ground truth standard. It consists of 200 recipes for each of the following recipe categories: appetizers and snacks, breakfast and lunch, dessert, dinner, and drinks. The uncurated version consists of approximately 22,000 recipes, which are only annotated with the rule-based NER, without being checked by subject matter experts. The semantic tags used for annotations are taken from the Hansard corpus [32,45]. To the best of our knowledge, this is the first corpus with such annotated food entities.

### Food Semantic Resources

#### Hansard Corpus

The Hansard corpus [32] is part of the SAMUELS (Semantic Annotation and Mark-Up for Enhancing Lexical Searches) project, where semantic tags are organized in a hierarchy with 37 higher-level semantic groups. One of these groups is the *Food and Drink*, which is then split into 3 subcategories, that is, *food, production of food, farming,* and *acquisition of animals for food, hunting*. These have 125, 36, and 13 top-level semantic tags, respectively.

#### FoodOn Ontology

FoodOn is a farm-to-fork ontology about food, which supports food traceability [35]. It consists of information about food products, their sources, and information about preservation processes, packaging, etc. It is built to represent food-related entities and to provide vocabulary for nutrition, diet, and plant and animal agricultural rearing research. FoodOn interoperates with the Open Biological and Biomedical Ontology Library and imports material from several ontologies covering anatomy, taxonomy, geography, and cultural heritage. The ontology aims to cover gaps in the representation of food-related products and processes and is being applied to research and clinical data sets in the academia and government.

#### SNOMED CT Ontology

SNOMED CT is the most comprehensive multilingual clinical health care terminology [36]. It is a machine-readable collection of medical terms, where synonyms and clinical definitions are available for each of the codes. It consists of information about drugs, disorders, symptoms, diagnoses, procedures, body structures, food, and other concepts that are related to health care.

#### FoodOntoMap

FoodOntoMap is a recently published resource that is developed by using the FoodBase corpus [38]. It provides data normalization of the food entities according to different semantic resources. Specifically, for each extracted entity presented in the FoodBase corpus, the semantic tags from Hansard, FoodOn, OntoFood, and SNOMED CT are available. It is important to note that the semantic tags from resources other than Hansard are not available for some of the extracted food entities since they do not exist in the respective food ontologies themselves. The food entity coverage per semantic resource is presented by Popovski et al [37].

### BERT

BERT is a word representation model that achieves state-of-the-art results in many NLP tasks [10]. The main idea of BERT is the bidirectional training of the transformer, which is different from previously published models that were trained using just a text sequence either from left to right or from right to left. Many models predict the next word in a sequence, while BERT uses a masked model, which predicts words masked in random order. It is used for bidirectional representation learning. BERT follows the idea and value of transfer learning [46,47], starting with pretraining a representation language model and then performing fine-tuning of the model for a new learning task (eg, NER, Question Answering). The same architectures are used in the pretraining and the fine-tuning step. The only difference is in the output layers. The parameters from the pretrained model are used as initial parameters, which are further fine-tuned concerning the learning task that is being solved in the fine-tuning.

#### Pretraining of BERT

In this phase, we did not pretrain a BERT model on our corpus. Instead, we used 3 previously pretrained and publicly available BERT models to fine-tune them for the food NER task. Specifically, the 3 BERT models that were used were the original pretrained BERT model [10], the pretrained BioBERT standard model [15], and the BioBERT large model [15]. The original BERT model was trained on the BookCorpus with around 800 million words [48] and the English Wikipedia with around 2500 million words, from which only the texts were used, ignoring the headers, tables, and lists.

The BioBERT was trained to improve the model for tasks in the biomedical domain since the domain consists of a large number of domain-specific proper nouns and terms, which do not appear in normal texts. Different combinations of corpora were experimentally used for pretraining BioBERT. The combinations involved the following corpora: the BookCorpus and the English Wikipedia (same as the BERT model), PubMed abstracts with around 4500 million words, and PubMed Central full-text articles with around 13,500 million words. Finally, the model pretrained on the combination using the BookCorpus, the English Wikipedia, and PubMed abstracts using the BERT-base cased code provided by Google is known as the BioBERT language representation model (ie, BioBERT Standard). The same combination trained using the BERT-large cased code provided by Google is known as BioBERT large.

#### Fine-Tuning BERT

To perform food NER, we fine-tuned the original BERT and the 2 versions of the BioBERT model. In all the cases, for each class, we used the IOB (inside, outside, and beginning) tagging [49] prediction, which is a common tagging format in computational linguistics. In this process, we used the FoodBase corpus as the ground truth. However, this corpus may contain multiple Hansard tags for each food phrase, and we used a few methods for selecting the most representative tag for each phrase.

The fine-tuning was performed for the following tasks:

1. Food classification: This was performed for distinguishing food versus nonfood entity. In this task, we labeled all food phrases annotated in FoodBase with the tag FOOD and used this data set for training and validation.
2. Hansard parent: This was performed for distinguishing 48 classes from the Hansard corpus. In this task, we selected parent semantic tags from the Hansard hierarchy that correspond to the food phrases in FoodBase. In cases with multiple different parent tags present for the food phrase, we selected the first occurring parent.
3. Hansard closest: This was performed for distinguishing 92 classes from the Hansard hierarchy. In this task, for each food phrase in FoodBase, we chose the closest Hansard tag to the food phrase being annotated. The closest tag was selected using the minimum cosine distance between the BERT embedding of the food phrase and the BERT embeddings of the Hansard tag labels.
4. FoodOn: This was performed for distinguishing 205 classes, where the classes are semantic tags from the FoodOn ontology. For each food phrase in FoodBase, we selected the corresponding FoodOn class based on the FoodOntoMap mappings [40].
5. SNOMED CT: This was performed for distinguishing 207 classes, where the classes are semantic tags from the SNOMED CT ontology. In this task, we also used FoodOntoMap [40] to obtain the SNOMED CT class for the food phrase.

**Figure 3:**
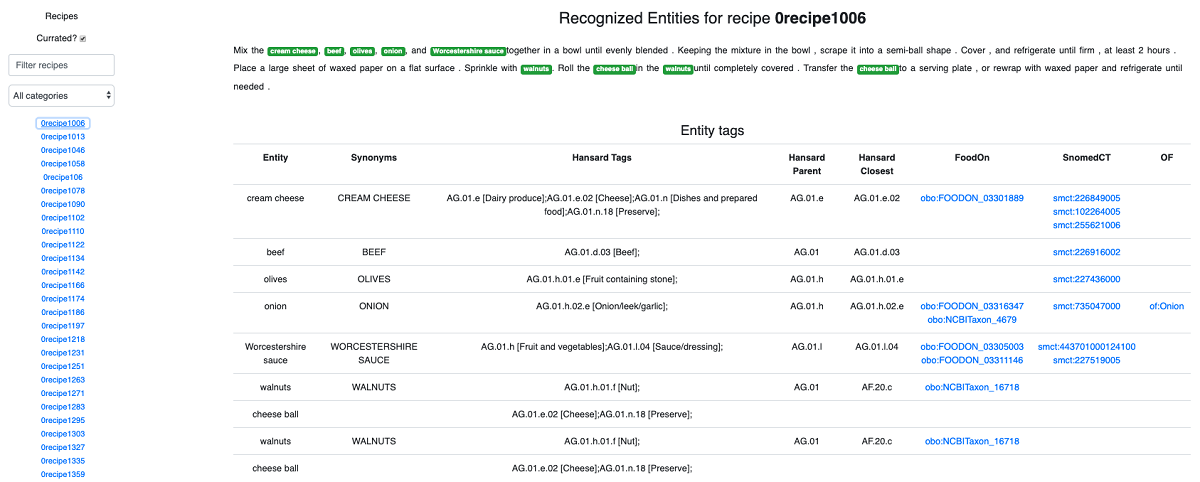
An example of food entities available from one recipe that are present in the training data set. The entities are annotated using Hansard parent, Hansard closest, FoodOn, Systematized Nomenclature of Medicine Clinical Terms, and OntoFood (not studied in this paper) semantic tags.

In cases of food versus nonfood entity task and the task of distinguishing food entities with regard to the Hansard semantic tags, we have a ground truth corpus—the curated part of FoodBase. However, in case of FoodOn and SNOMED CT, we fine-tuned BERT and BioBERT only for entities that had semantic tags provided by the FoodOntoMap resource (ie, not all food entities are presented in these 2 resources as was previously explained). All semantic tags (ie, Hansard parent, Hansard closest, FoodOn, and SNOMED CT) for each food entity available in the FoodBase corpus are presented by the FoodViz tool (see Figure 3). Finally, we ended up with 15 different fine-tuned models, 3 per task depending on the pretrained model that was used (BERT, BioBERT Standard, or BioBERT large).

#### A Baseline for Comparison: BuTTER

To compare the results, the Bidirectional Long Short-Term Memory (LSTM) model for sequence tagging with a CRF layer (BiLSTM-CRF) [50] was used as a baseline, which has already been shown to achieve state-of-the-art results in several NLP tasks such as part-of-speech tagging, chunking, and NER tasks. Additionally, the BiLSTM-CRF model has been used to train food NER (food versus nonfood entity) utilizing the food annotations available in the FoodBase corpus [38], resulting in BuTTER models. BuTTER consists of 2 different BiLSTM-CRF architectures, each one evaluated with 3-word embedding methods (ie, GloVe [51], Word2Vec [52], and FastText [53]) and once using the word tokens for representing the textual data used by the input layer. The difference between the 2 BuTTER architectures is that the first one is a BiLSTM-CRF model without character embeddings, while the second one has an additional stacked input and embedding layer to generate character embeddings (Char-BiLSTM-CRF). When representing the textual data using the predefined vocabularies of Word2Vec, GloVe, and FastText, some of the words are absent; therefore, out-of-vocabulary word preprocessing techniques can be applied to handle them. In the case when word tokens are used, the impact of lemmatization on the model performance was investigated. All in all, results from 16 different BuTTER models were obtained, that is, 2 architectures × 4 textual representations (ie, 3-word embeddings + word tokens × 2 scenarios, that is, preprocessing applied or not). More details about them can be found in the study of Comeau et al [39].

## Results

### Experiments

In this section, the experimental setups for fine-tuning the BERT and BioBERT models in each classification task are explained, followed by the experimental results obtained by the evaluation. We performed 2 experiments: (1) comparison of the BERT models with the corpus-based BuTTER models presented in a previous study [40] on the food versus nonfood entity task, and (2) presenting results for BERT models that can distinguish between different food semantic tags.

### Experimental Design

**Figure 4:**
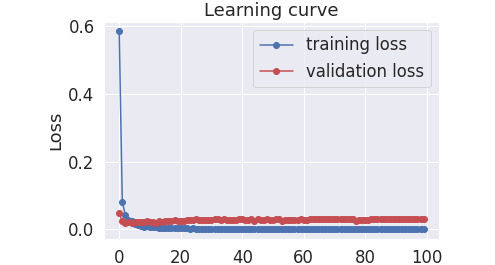
Training and validation loss per fine-tuning epoch for the bio bidirectional encoder representations from transformers large model on the Hansard parent data set.

The experiments were performed using the Colab platform [54]. To fine-tune the pretrained BERT and BioBERT models, HuggingFace’s *transformers* [55] library was used with its *BertForTokenClassification* class for token level prediction. This class wraps the *BertModel* class and adds a token-level classifier on top of it, which is a linear layer that takes the last hidden layer of the wrapped model as input. During the training of the fine-tuning, the *AdamW* optimizer was used with a *weight_decay_rate* of 0.01. The model was trained until its validation loss did not improve in 5 consecutive epochs, with a maximum of 100 epochs and with a scheduler to linearly reduce the learning rate throughout the epochs. Figure 4 presents the train and validation loss per fine-tuning epoch for the BioBERT large model on the Hansard parent data set. The same pattern holds for the other models, and therefore, we present the learning curve only for this particular model [56].

For the BiLSTM-CRF model architecture or the BuTTER models, we used the default parameters presented in the study of Comeau et al [39], which are also presented here:

1. The maximum sequence length (ie, sentence length) is 50 since the longest sentence in the data set consists of 45 tokens.
2. The batch size is 256.
3. Architecture: input layer with 50 units, embedding layer with 300 units, BiLSTM layer with 50 units (total of 100 parameters), dense (TimeDistributed) layer with 50 units, CRF output layer where the final output dimension is the number of classes + 1 (ie, one for padding).

The aforementioned architecture refers to the complete architecture of the BuTTER BiLSTM-CRF model, that is, the model without character embeddings. The BuTTER Char-BiLSTM-CRF model contains an additional stack of input and embedding layers for generating the character embeddings and a concatenation layer for concatenating the word embeddings with the character embeddings. The additional input layer contains 18 units, while the additional embedding layer contains 20 units. Each of the BuTTER models was trained until the improvement in validation loss of 5 consecutive epochs did not surpass 5*10^-3^, to a maximum of 1000 epochs, whichever comes first.

The data sets used for training and testing are from the curated version of FoodBase [38] transformed in IOB tagging [49] format [57]. The train portion contains 81,347 tokens, while we report the results with the remaining 25,828 tokens, that is, approximately 75% of the data is used for training and the rest is for testing of the model. The curated version of FoodBase contains 1000 recipes, with 5 categories that contain 200 recipes each. We use 150 recipes with alphabetically smaller identifiers of each category for training and the rest of the recipes from the category for testing. The statistics about the number of tokens and their classes among the different data sets are shown in Table 1. The “Number of different inside, outside, and beginning annotations” row in this table describes the classes that our model tries to predict. Since we are predicting food phrases, for each different food phrase class, we may have annotations that start with *B-* for the first token in the phrase, and *I-* for all the rest of the tokens. Therefore, the number of different IOB annotations is approximately twice as large comparing it to the number of phrase classes. Additionally, the data sets are not balanced since the majority of the tokens are not part of the food phrase, that is, they are outside tokens. The *Hansard parent* data set is smaller than the others since there were 4 recipes with problematic parents and we omitted them in the evaluation.

**Table 1:** Data set statistics.

Annotations	Food classification	Hansard parent	Hansard closest	FoodOn	SNOMED CT^a^
Annotated tokens (beginning and inside)	17,937	11,759	17,864	8730	8151
Outside tokens	95,416	95,416	88,956	98,445	99,024
Number of different inside, outside, and beginning annotations	3	63	163	342	318
Number of food phrase classes	1	34	91	197	196
Total number of tokens	107,175	107,176	106,820	107,175	107,175

^a^SNOMED CT: Systematized Nomenclature of Medicine Clinical Terms.

The evaluation of the proposed models was done using stratified five-fold cross-validation. Stratified sampling was used to generate the folds since the FoodBase corpus consists of 5 different categories of recipes. For each recipe category, 10% of the training set of each fold was taken sequentially out and used for validation.

### Experimental Results

Next, the results for both experiments are presented, starting with the comparison of the BERT models with the BuTTER models on the food versus nonfood task, followed by presenting the BERT models trained for distinguishing between different food semantic tags. We present the results for the macro F1 score. The macro averaging scheme computes each metric for each class independently and then calculates the mean. The rationale behind using macro averaging is that it conveys more meaningful information when considering especially a task that consists of more than two semantic tags that should be predicted with heavily unbalanced data. Conversely, simple micro averaging provides insufficient information in tasks where more than two semantic tags (ie, classes) are used, as it conflates the true positives, false positives, true negatives, and false negatives into one confusion matrix and then computes the evaluation metrics. Similarly, weighted averaging is biased in favor of the class most represented in the data, as the weight while computing the average depends on the relative frequency of the class label in the data set.

### Comparison With the BuTTER Approach

**Figure 5:**
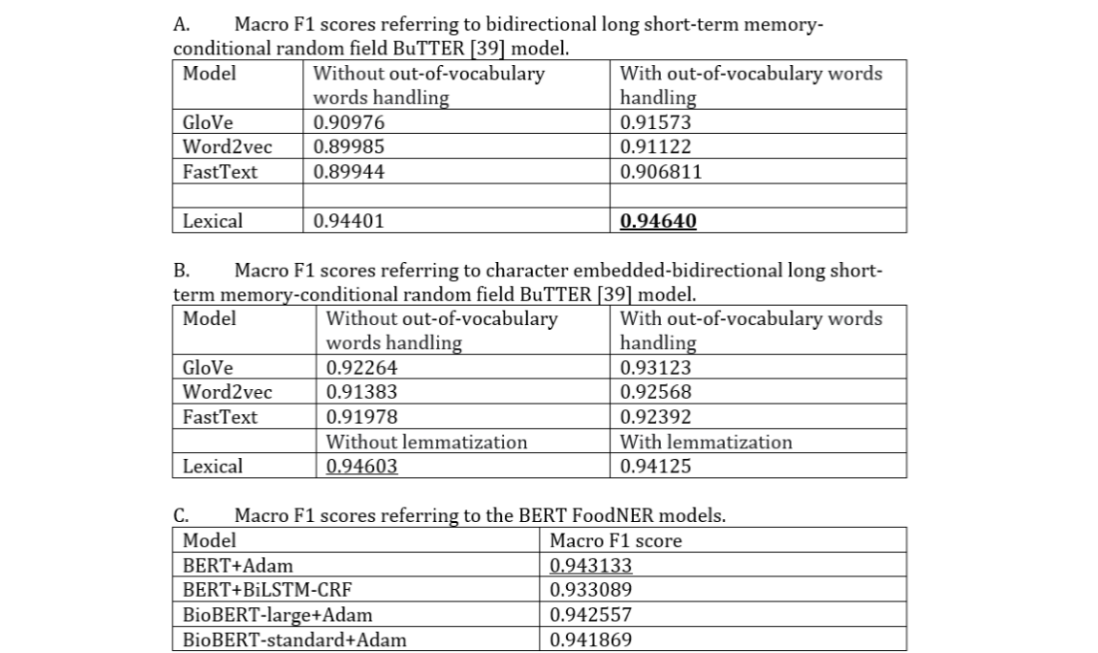
Macro F1 scores for all considered models for the food versus nonfood entity task. Each macro F1 score is obtained by using stratified k-fold cross-validation (k=5). Underlined values are best per subtable, while the bold value is the best from the whole table. BERT: bidirectional encoder representations from transformers; BiLSTM-CRF: bidirectional long short-term memory conditional random field; BuTTER: bidirectional long short-term memory for food named-entity recognition; NER: named-entity recognition.

**Figure 6:**
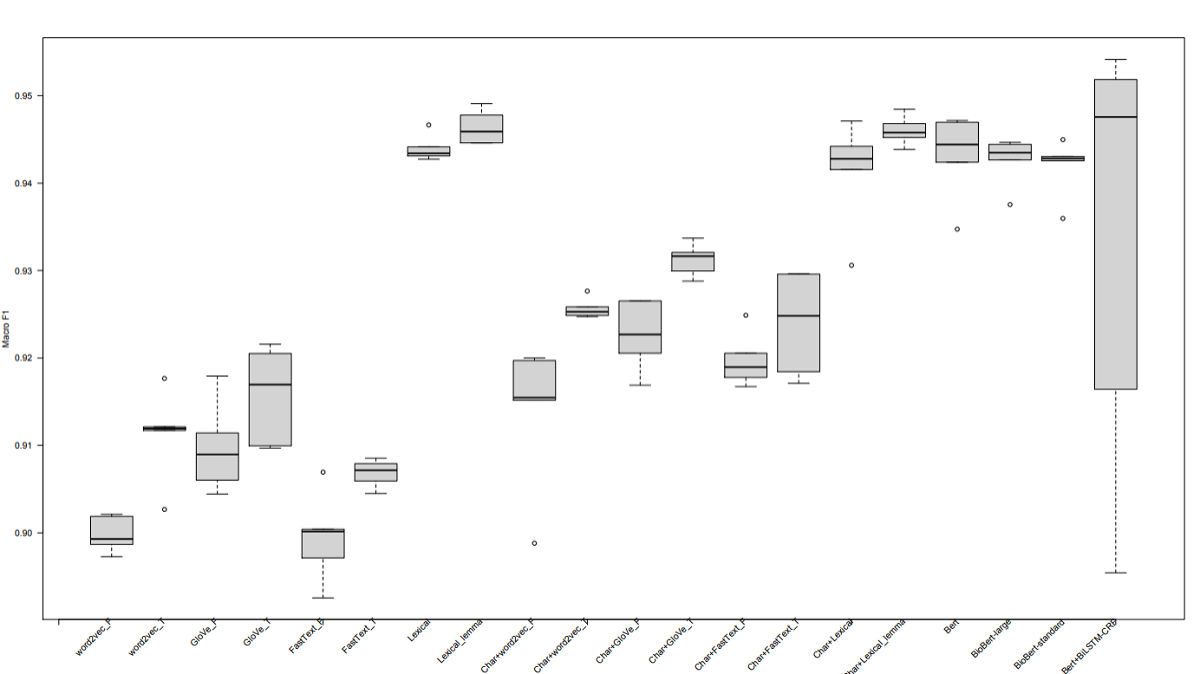
Boxplots of macro F1 scores obtained by using stratified five-fold cross-validation for all considered models for the binary food classification task. BERT: bidirectional encoder representations from transformers; BiLSTM-CRF: bidirectional long short-term memory conditional random field.

Figure 5 [39] presents the results obtained from evaluating the fine-tuned BERT (ie, FoodNER) by using the original pretrained BERT model and 2 BioBERT models in the food versus nonfood task described in Methods and comparing them with the BuTTER results obtained for the same task. From the table, it follows that the best FoodNER model is obtained by fine-tuning the original pretrained BERT, resulting in a macro F1 score of 94.31%. Additionally, comparing it with the other FoodNER models obtained by fine-tuning BioBERT large and BioBERT standard, the absolute empirical differences are very small, amounting to only 0.05% and 0.12%, respectively. Comparing the FoodNER models with both BuTTER architectures (BiLSTM-CRF and Char-BiLSTM-CRF) when word embeddings are used to represent the textual data for the input layer (ie, GloVe, Word2Vec, and FastText), it follows that all FoodNER models have better macro F1 scores by using the stratified 5-fold cross-validation. However, we should point that the differences here are in the range from 1.74% to 4.36%. Comparing FoodNER models with both BuTTER architectures when word tokens are used for the input layer, the BiLSTM-CRF with lemmatization of the word tokens outperforms the FoodNER models by 0.32% and the Char-BiLSTM-CRF without lemmatization of the word tokens by 0.28%. We can conclude here that these differences are not crucial from a practical point of view; therefore, we can assume that all models perform similarly. Further, we also fine-tuned BERT by using the BiLSTM-CRF architecture for food classification, which results in a similar performance of a macro F1 score of 93.30%. To explore the robustness of the models, Figure 6 presents boxplots of the macro F1 score distributions obtained by evaluating each fold for each model separately. From the figure, it follows that all models perform well since all of them provide a macro F1 score greater than 87.00% for each fold. The most robust models are FoodNER BioBERT standard model and the BuTTER BiLSTM-CRF model with Word2Vec when out-of-vocabulary preprocessing is applied. However, comparing the results between both models, the FoodNER BioBERT standard provides a better macro F1 score. The other models also provide robust results, where the macro F1 scores obtained from different folds do no vary with large deviations. It is interesting to note that the best macro F1 score is obtained when BERT is fine-tuned with BiLST-CRF for one of the five folds; however, using the values from the other folds, the macro F1 score of this model can vary between different folds. Thus, we can conclude that the FoodNER models, which are fine-tuned BERT, BioBERT standard, and BioBERT large models, provide very robust results. These results also show that by using BERT, state-of-the-art results for food classification can be achieved.

### BERT Models for Recognizing Between Different Food Semantic Tags

In this experiment, we present the results of fine-tuning the BERT, BioBERT large, and BioBERT standard models in the tasks of distinguishing food entities concerning different semantic models (ie, FoodOn, Hansard closest, Hansard parent, and SNOMED CT). We have decided to focus only on the BERT models since it provides state-of-the-art results in already all NLP NER tasks. Additionally, in Table 1, the number of annotated tokens and the number of classes for each task are presented.

Table 2 provides the macro F1 scores for the 3 FoodNER models (BERT, BioBERT large, and BioBERT standard) for distinguishing food entities concerning different semantic models (ie, FoodOn, Hansard closest, Hansard parent, and SNOMED CT). The column *“epochs”* provides information for the number of epochs needed to fine-tune the model. From the table, it is evident that all models achieved a macro F1 score between 73.39% and 78.96%. The best models for each semantic tag set achieved the following macro F1 scores: (1) FoodOn, 78.13%; (2) Hansard closest, 78.96%; (3) Hansard parent, 76.26%; and (4) SNOMED CT, 76.01%.

Keeping in mind the number of classes we are predicting for each task, we can conclude that these are really promising results. Additionally, the FoodNER models trained in the tasks of distinguish food entities concerning semantic tags on the level of food groups are the first corpus-based NERs that can distinguish between different food semantic tags (ie, food groups). Once more, we should emphasize that in the cases of FoodOn and SNOMED CT, the BERT and BioBERT models are tuned only on the entities that have semantic tags provided by the FoodOntoMap resource, in which not all food entities from the semantic resources are present.

**Table 2:** Macro F1 scores for the 3 food named-entity recognition models for the tasks concerning different semantic models.

Model, semantic model			Epochs^a^	Macro F1 score (%)	
**BERT^b^**					
	FoodOn	100			78.13
	Hansard closest	85			75.87
	Hansard parent	100			75.04
	SNOMED CT^c^	91			76.01
**BioBERT-large**					
	FoodOn	93			75.58
	Hansard closest	100			78.96
	Hansard parent	100			76.26
	SNOMED CT	95			74.51
**BioBERT-standard**					
	FoodOn	100			74.81
	Hansard closest	100			74.18
	Hansard parent	89			74.94
	SNOMED CT	89			73.39

^a^This provides information on the number of epochs needed to fine-tune the model.

^b^BERT: bidirectional encoder representations from transformers.

^c^SNOMED CT: Systematized Nomenclature of Medicine Clinical Terms.

## Discussion

### Principal Findings

**Figure 7:**
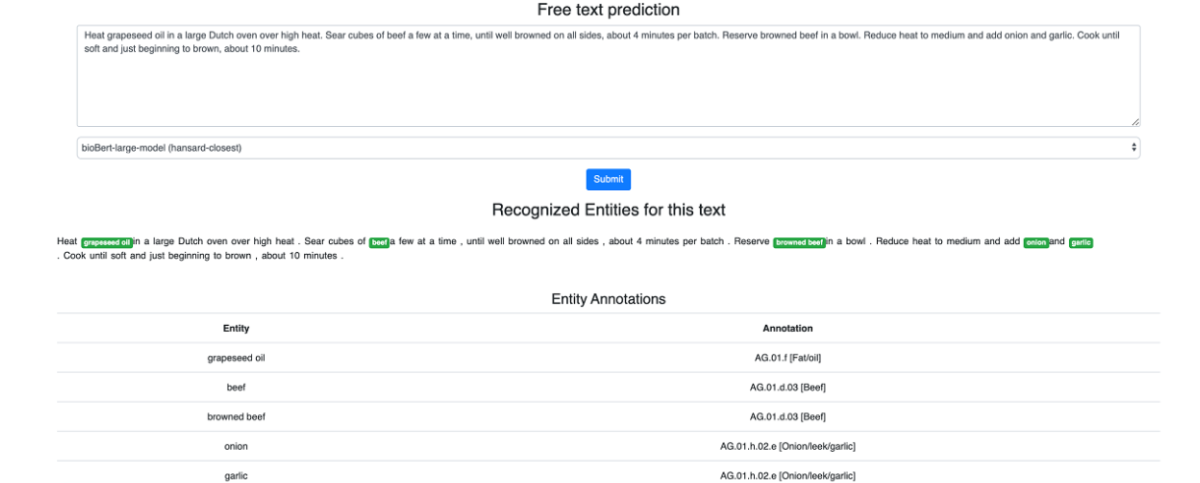
Food named-entity recognition integration in FoodViz.

The models are trained on FoodBase [38], in which recipes that are collected from the biggest social media networks for sharing and discovering recipes, are annotated. Since this is a specific type of text, there are some weaknesses when it comes to applications on texts of a different nature (eg, medical texts). To address this in our future work, we plan to further retrain the models on various types of documents such as dietary recommendations and PubMed articles. Regardless of this, the presented BERT models are robust for extracting food concepts while simultaneously normalizing them to some semantic resource, which allows further interlinking of the entities with other domains (eg, health and environmental sciences). This will help to improve the quality of health and clinical practices. The semantic tags were selected based on the food annotations that exist from the FoodBase and FoodOntoMap resources. However, in future, the FoodNER methodology may be applied on any other annotated corpus from this domain. To bring our work closer to subject matter experts from the food domain, the FoodNER models have been integrated in the FoodViz platform [45]. Figure 7 shows the interface where subject matter experts can place an arbitrary recipe, select a model, and preview the annotated food entities. We provide highlighting of the phrases in the text, as well as the tabular display of the food phrases and their annotations. Figure 7 is an example where a short description from a recipe *“Heat rapeseed oil in a large Dutch oven over high heat. Sear cubes of beef a few at a time, until well browned on all sides, about 4 minutes per batch. Reserve browned beef in a bowl. Reduce heat to medium and add onion and garlic. Cook until soft and just beginning to brown, about 10 minutes.”* is annotated using the model fine-tuned with BioBERT large in the Hansard closest task. From the annotations provided, it is obvious that the model can recognize all food entities that are mentioned in the text (ie, *grapeseed oil, beef, browned beef, onion,* and *garlic*) annotated by Hansard semantic tags. This interface radically simplifies the usage of the state-of-the-art models for subject matter experts in the food domain, without their knowledge of the underlying details, such as machine learning or IOB format understanding. Additionally, the current architecture of the FoodViz application allows integration of new prediction models only with their upload at the corresponding location in the server.

### Conclusion

We present a corpus-based NER method for food information extraction, known as FoodNER. It is developed by fine-tuning the BERT model by using 3 previously published predefined BERT representation language models (ie, the original BERT and 2 BioBERTs; standard and large). FoodNER can be used to extract and annotate food entities in 5 different tasks: distinguishing between food versus nonfood entities and distinguishing food entities on the level of food groups by using the closest Hansard semantic tags, the parent Hansard semantic tags, the FoodOn semantic tags, or the SNOMED CT semantic tags. All in all, the models provide very promising results achieving around 93.30%-94.31% macro F1 scores in the food versus nonfood entity task and around 73.39%-78.96% macro F1 scores in the tasks where more semantic tags are recognized. Additionally, the models are included in the FoodViz framework, which allows users to select which FoodNER model they want to use for the annotation of their texts with food entities and additionally provides a visualization of the annotated data with an opportunity to correct the false positive and false negative annotations. Having such a robust state-of-the-art food information extraction method such as FoodNER will allow further research in investigating food-drug and food-disease interactions, thereby providing an opportunity to start building a food knowledge graph, including relations with health-related entities.

## Abbreviations

BERT: bidirectional encoder representations from transformers
BiLSTM-CRF: bidirectional long short-term memory conditional random field
BuTTER: bidirectional long short-term memory for food named-entity recognition
IOB: inside, outside, and beginning
NCBO: National Center for Biomedical Ontology
NER: named-entity recognition
NLP: natural language processing
SNOMED CT: Systematized Nomenclature of Medicine Clinical Terms
